# The impact of taxane-based preoperative chemotherapy in gastroesophageal signet ring cell adenocarcinomas

**DOI:** 10.1186/s13045-015-0148-y

**Published:** 2015-05-15

**Authors:** Stefano Kim, Frederic Fiteni, Sophie Paget-Bailly, François Ghiringhelli, Zaher Lakkis, Marine Jary, Francine Fein, Franck Bonnetain, Christophe Mariette, Christophe Borg

**Affiliations:** Department of Medical Oncology, University Hospital of Besancon, 3 Boulevard Alexander Fleming, Besancon, F-25030 France; Clinical Investigational Centre, CIC-1431, University Hospital of Besançon, Besançon, France; INSERM, Unit 1098, University of Franche-Comté, Besançon, France; Methodology and Quality of Life in Oncology Unit, University Hospital of Besançon, Besançon, France; Department of Oncology, Georges-Francois Leclerc Cancer Center, Dijon, France; Department of Digestive Surgery and Liver Transplantation, University Hospital of Besançon, Besançon, France; Department of Gastroenterology, University Hospital of Besançon, Besançon, France; EA 3181 University of Franche-Comté, Besançon, Besançon, France; Department of Digestive Surgery, Lille University Hospital, Lille, France

**Keywords:** Signet ring cell, Gastroesophageal cancer, Gastric cancer, Preoperative, Neoadjuvant, Taxane, Docetaxel, Paclitaxel

## Abstract

The benefit of preoperative chemotherapy in resectable gastroesophageal adenocarcinomas was not observed in signet ring cell subtype. However, the potential interest of taxane-based preoperative chemotherapy on this subtype is still an unresolved issue. Nineteen patients with localized signet ring cell adenocarcinomas received taxane-based regimens, and 17 patients underwent surgery. Complete resection was achieved in 80 %, and median overall survival was 40.8 months (95 % confidence interval (CI), 20.2—not reached). Even though one patient achieved a complete pathological response, seven patients had an upstaging of their tumors at surgery. The potential benefits of taxane-based chemotherapy seem to be limited to a reduced number of patients.

**Electronic supplementary material:**

The online version of this article (doi:10.1186/s13045-015-0148-y) contains supplementary material, which is available to authorized users.

## Findings

### Background

Signet ring cell (SRC) adenocarcinoma is a particular histological subtype of gastroesophageal adenocarcinomas (GEA) displaying a worse prognosis [1]. Even though the perioperative chemotherapy (PCT) in resectable GEA demonstrated a significant benefit in terms of overall survival (OS) compared to surgery alone [2, 3], this benefit seems to be limited to non-SRC histology [4]. This observation prompted physicians to perform surgery without preoperative chemotherapy in SRC GEA patients with a resectable disease.

Taxanes are potent microtubule-stabilizing agents with demonstrated antitumor activity in advanced GEA and with encouraging results in resectable GEA, as reported in several phase II trials [5–10]. The potential interest of taxane-based PCT on SRC GEA is still an unresolved issue.

## Results

Between January 2005 and December 2012, 19 patients with localized SRC GEA received taxane-based PCT from six French hospitals. (Additional file 1) Patients’ median age was 64 years (range, 41–81 years). The majority of tumors (58 %) were located in the stomach and were predominantly stage III (42 %) and II (42 %) (Table 1).

**Table 1:** Patient and tumors’ characteristics

Patient and tumors’ characteristics before surgery		
	*N* = 19	
Age, years (range)	64	41–81
Gender	No	%
Male	14	74
Female	5	26
ECOG-PS	No	%
0	11	58
1	5	26
2	3	16
Location of tumor	No	%
Distal esophagus or GEJ	8	42
Stomach	11	58
Clinical TNM stage	No	%
Stage I	3	16
Stage II	8	42
Stage III	8	42
Stage IV	0	0
Neoadjuvant chemotherapy	No	%
DCF	7	37
PET	7	37
TFOX	3	16
Docetaxel-Cisplatin	1	5
Cisplatin-Paclitaxel-Doxorubicin	1	5
Surgery and postoperative variables		
	*N* = 17	
Type of surgery, No (%)	No	%
Total gastrectomy	8	47
Subtotal gastrectomy	2	12
Lewis–Santi esophagectomy*	7	41
Lymphadenectomy extend	No	%
D1	4	31
Modified D2	6	46
D2	3	23
Missing	4	–
Resection	No	%
R0	12	80
R1	3	20
R2	0	0
Missing	2	–
Pathological tumor classification	No	%
pT0	1	6
pT1	2	13
pT2	3	19
pT3	5	31
pT4	5	31
Missing	1	–
Pathologic nodal classification	No	%
pN0	2	13
pN1	6	38
pN2	3	19
pN3	5	31
Missing	1	–
Pathologic metastatic stage	No	%
pM0	15	88
pM1	2	12
Adjuvant treatment	No	%
DCF	3	18
Docetaxel-Cisplatin	1	6
TFOX	1	6
mFOLFOX6	6	35
EOX	2	12
Chemoradiotherapy	2	12
No adjuvant treatment	2	12

*ECOG-PS* Eastern Cooperative Oncology Group—performance status, *GEJ* gastroesophageal junction, *DCF* 3 cycles of docetaxel (75 mg/m^2^ d1), cisplatin (75 mg/m^2^ d1), and 5-fluorouracil (750 mg/m^2^/d on continuous perfusion on days 1 to 5), every 3 weeks, *PET* 8 cycles of cisplatin (30 mg/m^2^ d1), epirubicin (50 mg/m^2^ d1), and paclitaxel (80 mg/m^2^ d1), every week, *TFOX* docetaxel (50 mg/m^2^), oxaliplatin (85 mg/m^2^), leucovorin (400 mg/m^2^) and 5FU continuous infusion 48 h (2400 mg/m^2^), *S* surgery alone, *D1* lymphadenectomy limited to regional lymph nodes, *modified D2* extended lymph node dissection without pancreatectomy and splenectomy, *D2* extended lymph node dissection with pancreatectomy and/or splenectomy, *mFOLFOX6* oxaliplatin, leucovorin, 5FU bolus and 5FU continuous infusion 48 h, *EOX* epirubicin, oxaliplatin and capecitabine

*Oesophagectomy via abdominal and right thoracic approaches

Most frequent neoadjuvant chemotherapy regimens were DCF regimen in seven patients (37 %), as described in TAX-325 trial, and PET regimen in other seven patients (37 %) as previously published [5, 10]. Three patients (16 %) received TFOX regimen (Table 1).

Seventeen patients (89 %) underwent surgery. One patient presented an unexpected death (cardiac failure) after three DCF cycles and before surgery, and another patient refused surgery after eight PET cycles. Total gastrectomy was performed in eight patients (47 %) and esophagogastrectomy via abdominal and right thoracic approaches (Lewis–Santi) in seven patients (41 %). Postoperative adverse events were observed in three patients with favorable recovery (Table 1).

All 17 patients who underwent surgery had a curative-intent resection. Pathological information about surgical margins was available in 15 patients, and the pathological complete resection (R0) was achieved in 12 patients (80 %). One patient presented a complete pathological response (pCR). This patient had a T2 disease with lymph node enlargement at diagnosis. In seven patients, more advanced disease was found at surgery compared to initial staging. Two patients presented intraoperative peritoneal metastases, and five patients had T4 disease (Table 1).

**Fig. 1:**
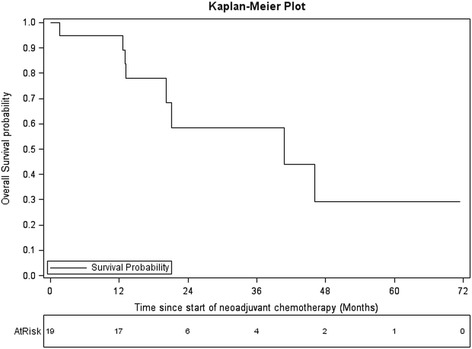
OS estimated using Kaplan Meier method

After a median follow up of 26.2 months (15.5–71.5), eight patients died and nine patients progressed. The median OS was 40.8 months (95 % confidence interval (CI), 20.2—not reached), and the median PFS was 36.8 months (95 % CI, 10.0—not reached). Five-year OS and PFS rates were 30.4 % and 29.3 %, respectively (Fig. 1).

## Conclusion

Even though our study has obvious limitations as a retrospective analysis and regarding the limited number of patients, this is the largest cohort of SRC GEA patients treated with preoperative taxane-based chemotherapy published so far. The potential benefits of taxane-based PCT seem to be limited to a reduced number of patients with SRC GEA. The high number of patients with pathological upstaging reinforces the results of Messager et al. and the recommendation to perform front line surgery in resectable SRC GEA without PCT [4]. Future efforts should be focused on developing predictive biomarkers to identify SRC GEA patients potentially sensitive to taxanes.

### Future perspectives

Targeted agents have shown promising results in advanced GEA. [11] Among them, trastuzumab (in HER2 positive patients) and ramucirumab have been approved in advanced GEA. However, most SRC GEAs are HER2 negative, and ramucirumab, an antiangiogenic mAb selectively targeting VEGFR2, will hardly be developed in perioperative setting due to negative experience of bevacizumab in gastrointestinal adenocarcinomas [12, 13]. Among novel molecules in development in GEA, checkpoint inhibitors are probably the most promising. Pembrolizumab, an antiPD1 mAb was administered as monotherapy in 39 GEA patients with PD-L1 expression. Most patients have received ≥2 prior chemotherapies. An encouraging overall response rate of 22 % and the 6-month OS rate of 69 % were observed [14]. The expression of PD-L1 in SRC GEA is present in about 23 %, and a growing body of evidence suggests that taxane induces immunogenic cell death sustaining the potential interest to combine taxane and antiPD1 in clinical trials including SRC GEA patients [15, 16].

Pembrolizumab and other checkpoint inhibitors should be evaluated in prospective preoperative trials in GEA patients including SRC histology, probably in association with taxane-based chemotherapy. Future exhaustive molecular analysis in SRC GEA is needed to find targets for novel molecules in this chemorefractory disease.

## Additional file

Additional file 1:
**Methods.**

## Abbreviations

SRC: Signet ring cell
GEA: Gastroesophageal adenocarcinomas
PCT: Preoperative chemotherapy
OS: Overall survival
DCF: 3 cycles of docetaxel, cisplatin, and 5-fluorouracil, every 3 weeks
PET: 8 cycles cisplatin, epirubicin, paclitaxel, weekly
TFOX: 6 cycles of docetaxel, oxaliplatin, leucovorin, and 5FU, every 2 weeks
R0: Complete resection
pCR: Pathological complete response
PFS: Progression free survival
HER2: Human epidermal growth factor receptor 2
mAb: Monoclonal antibody
VEGFR2: Vascular endothelial growth factor receptor 2
PD1: Programmed cell death protein 1
PD-L1: Programmed cell death-ligand 1
