# Supplementary material for: The impact of taxane-based preoperative chemotherapy in gastroesophageal signet ring cell adenocarcinomas
Source: J Hematol Oncol. 2015 May 15;8:52. doi: 10.1186/s13045-015-0148-y (PMC4440289; doi:10.1186/s13045-015-0148-y)
Supplement: Additional file 1: — Methods. [file 13045_2015_148_MOESM1_ESM.docx]

**Methods**

The aim of our retrospective exploratory study was to evaluate the potential interest of taxane-based preoperative chemotherapy in signed-ring cell subtype in locally advanced GEA patients. Database from 6 French hospitals were studied comprising University Hospital of Besançon, Nord Franche-Comté Hospital, Lille University Hospital, Georges-Francois Leclerc Cancer Center at Dijon, Hospital of Vesoul, and Polyclinic Franche-Comté hospital.

The primary endpoint was the interest of taxane-based preoperative chemotherapy in signed-ring cell gastroesophageal adenocarcinoma patients in terms of overall survival (defined as time interval between the first chemotherapy and death of all causes). The secondary endpoints were the complete resection rate, and the progression free survival defined as time interval between the first chemotherapy and the first progression (local, regional, distant and second cancer), death (from all causes) or data cut-off (from all causes). Overall survival and progression free survival were estimated using Kaplan-Meier estimation and described with 95 % confidence interval. Follow-up was calculated using reverse Kaplan-Meier estimation.
